# Effective elimination of liver cancer stem-like cells by CD90 antibody targeted thermosensitive magnetoliposomes

**DOI:** 10.18632/oncotarget.9116

**Published:** 2016-04-29

**Authors:** Rui Yang, Li Y. An, Qin F. Miao, Feng M. Li, Yong Han, Hui X. Wang, Dang P. Liu, Rong Chen, Sha Q. Tang

**Affiliations:** ^1^ School of Medicine, Southeast University, Nanjing, People's Republic of China; ^2^ Jiangsu Key Laboratory of Molecular and Fuctional Imaging, Department of Radiology, Zhongda Hospital, Nanjing, People's Republic of China; ^3^ Department of Oncology, Zhongda Hospital, Nanjing, People's Republic of China

**Keywords:** LCSCs, CD90, TMs, targeting therapy, hyperthermia therapy

## Abstract

**Aim:**

To investigate the use of thermosensitive magnetoliposomes (TMs) loaded with magnetic iron oxide (Fe_3_O_4_) and the anti-cancer stem cell marker CD90 (CD90@TMs) to target and kill CD90^+^ liver cancer stem cells (LCSCs).

**Methods:**

The hepatocellular carcinoma cell line Huh7 was used to separate CD90^+^ LCSCs by magnetic-activated cell sorting. CD90@TMs was characterized and their ability to target CD90^+^ LCSCs was determined. Experiments were used to investigate whether CD90@TMs combined with magnetic hyperthermia could effectively eliminate CD90^+^ LCSCs.

**Results:**

The present study demonstrated that CD90^+^ LCSCs with stem cells properties were successfully isolated. We also successfully prepared CD90@TMs that was almost spherical and uniform with an average diameter of 130±4.6 nm and determined that magnetic iron oxide could be incorporated and retained a superparamagnetic response. CD90@TMs showed good targeting and increased inhibition of CD90^+^ LCSCs *in vitro* and *in vivo* compared to TMs.

**Conclusion:**

CD90@TMs can be used for controlled and targeted delivery of anticancer drugs, which may offer a promising alternative for HCC therapy.

## INTRODUCTION

Hepatocellular carcinoma (HCC) is the third-leading cause of death worldwide [1], despite advances in cancer therapeutics. Liver cancer stem-like cells have been recognized in multiple subtypes of HCC and identified as a contributor to HCC initiation, relapse and metastasis [2]. CD90 is an important marker for liver cancer stem-like cells [3] found in all HCC cells and 91.6% of blood specimens from liver cancer patients [4]. A recent study on the relationship between liver cancer stem cells (LCSCs) and early recurrence of HCC indicated that early recurrence was related to expression of CD90 [5]. These studies suggest that CD90^+^ cells are important for HCC initiation, relapse and treatment. Moreover, CD90^+^ HCC cells, but not CD90^−^ HCC cells, caused tumor formation in immunodeficient mice. In addition, when gene expression was compared in CD90^+^ LCSCs and pericarcinomatous tissue, CD90^+^ HCC cells expressed genes that contributed to inflammation and drug resistance [6], suggesting that CD90 was a more sensitive and specific marker of liver cancer stem-like cells in HCC. So in our previous study, CD90^+^ liver cancer stem-like cells were referred to as CD90^+^ LCSCs. However, few treatments specifically target LCSCs, which may contribute to the poor prognosis of HCC patients. Thus, synthesizing a compound that can selectively scavenge CD90^+^ LCSCs for treatment of HCC cells would generate much interest.

Radiotherapy and chemotherapy have long been the conventional tumor treatment modalities. However, resistance reduces efficacy and often gives rise to recurrence. Recent studies demonstrated that increased expression of breast cancer resistance protein 1 (BCRP1) [7] and O (6)-methylguanine-DNA methyltransferase (MGMT) [8] were responsible for chemoresistance by cancer stem cells (CSCs). Another study showed that repair mechanisms in response to DNA damage by radiation caused radioresistance [9]. Therefore, developing novel approaches to eradicate CSCs shows promise for radical elimination of tumors.

Recent studies have focused on eradicating CSCs by magnetic hyperthermia because of its important roles in improving sensitivity to chemotherapy [10] and radiotherapy [11], as well as in overcoming drug resistance [12]. Sadhukha et al. [13] stated that CSCs could be eliminated by magnetic hyperthermia, while in fact CSCs exhibited increased tolerance than non-CSCs to radiotherapy and chemotherapy. This supports the investigation of magnetic hyperthermia as a new and more effective treatment for CSCs compared with radiotherapy and chemotherapy.

**Scheme 1:**
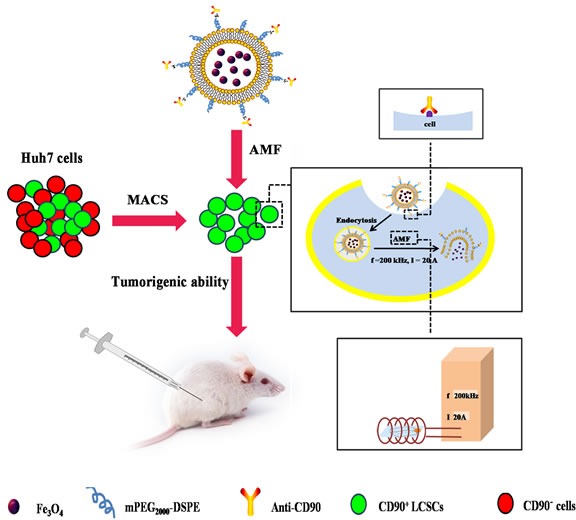
Effective elimination of LCSCs by CD90@TMs The TMs and CD90@TMs was prepared to target and kill CD90^+^ LCSCs. It was demonstrated that CD90^+^ LCSCs could be effectively ablated by CD90@TMs. Magnetic hyperthermia-treated CD90^+^ LCSCs could cause a significant delay in tumor initiation *in vivo* tumor initiation study. CD90@TMs showed higher inhibition rate of tumor mass and tumor volume compared to TMs in hepatocarcinoma-bearing mice. **Abbreviations:** CD90, cluster of differentiation 90; PEG_2000_-DSPE, 1,2-distearoyl-sn-glycero-3-phosphoethanolamine-N-[methoxy(polyethylene glycol) -2000]; TMs, thermosensitive magnetoliposomes; LCSCs, live cancer stem cells; AMF, alternating magnetic field; MACS, magnetic-activated cell sorting.

Despite these advantages, conventional hyperthermia therapy failed to alleviate toxicity due to dispersed heating of the adjacent organs and normal tissue [14]. To solve this problem, magnetic hyperthermia, which was initially proposed by Gilchrist, has been promoted as a tumor heat treatment that can precisely deliver heat to the site of action [15]. The temperature of the tissue can be controlled by an external magnetic field; therefore, no thermal damage to non-target zones occurs. Superparamagnetic Fe_3_O_4_ nanoparticles have considerable magnetism, catalysis, and wave absorption properties, making them the most commonly used magnetic fluid for tumor hyperthermia [16]. Fe_3_O_4_ loaded with drugs can enhance drug concentration in the target with the assistance of an external magnetic field to improve therapeutic tumor efficacy while reducing normal tissue toxicity. In addition, Fe_3_O_4_ can be used as a contrast agent in magnetic resonance imaging (MRI) to follow drug distribution. When placed in alternating magnetic field (AMF), it generates thermal energy that can be used to induce hyperthermia and control the release of drugs [17, 18]. However, a short half-life, lack of active targeting ability and removal by macrophages in the mononuclear phagocytic system (MPS) has limited its application [19]. To overcome these problems, liposomes have been loaded with Fe_3_O_4_ and polyethylene glycol (PEG) to facilitate membrane insertion. In this study, the thermal-sensitive lipid, dipalmitoylphosphatidylcholine (DPPC), was selected as a membrane material to enhance the controllability of Fe_3_O_4_ and efficacy against hyperthermia (Scheme 1). Thermal-sensitive lipsomes is an ideal approach for drugs when combined with hyperthermia. The release situation can be adjusted by the temperature. The drug release percents of the thermal-sensitive lipsomes are improved significantly when the temperature is higher than the phase transition temperature, while it releases less in non-heated organs [19].

To our knowledge, there are few reports describing the influence of magnetic hyperthermia for LCSCs and non-LCSCs. In this study, we successfully isolated CD90^+^ LCSCs and determined their sensitivity to magnetic hyperthermia. CD90 thermosensitive magnetoliposomes (CD90@TMs) was subsequently prepared to target CD90^+^ LCSCs and we explored whether CD90^+^ LCSCs could be effectively ablated by CD90@TMs (Scheme 1). *In vivo* tumor initiation study performed in mice showed a significant delay in tumor initiation with CD90@TMs mediated magnetic hyperthermia-treated cells compared to the controls. The results demonstrate for the first time that CD90@TMs facilitates drug delivery to LCSCs, and CD90@TMs mediated hyperthermia efficiently induced death of CD90^+^ LCSCs.

## RESULTS AND DISCUSSION

### Characterization of CD90@TMs

**Figure 1:**
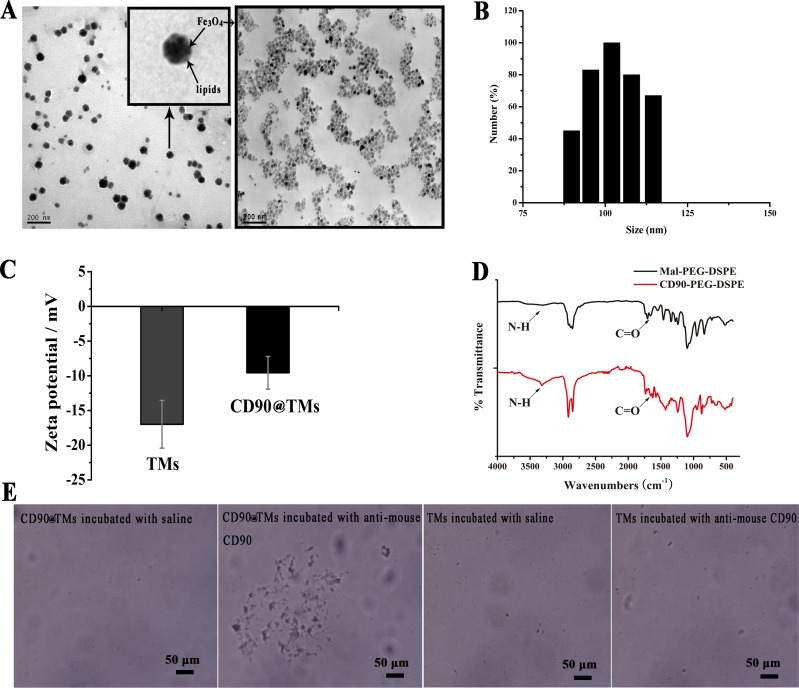
Characterization of CD90@TMs **A.** TEM image of Fe_3_O_4_ and CD90@TMs (The bar = 200 nm). **B.** Liposomes size determined by ZetaPlus. **C.** Zeta potentials determined by ZetaPlus (mean ± SD, *n* = 3). **D.** FTIR spectra of Mal-PEG_2000_-DSPE and CD90-PEG_2000_-DSPE. **E.** The slide agglutination method of CD90@TMs (The bar = 50μm). Abbreviations: TEM, transmission electron microscope; TMs, thermosensitive magnetoliposomes; FTIR, fourier translation infrared spectroscopy; PEG_2000_-DSPE, 1,2-distearoyl-sn-glycero-3-phosphoethanolamine-N-[methoxy(polyethylene glycol)-2000]; CD90, cluster of differentiation 90.

Liposome is a commonly used drug vector that facilitates drug targeting and delays release, while reducing the dose and drug toxicity [19]. However, the MPS can cause rapid elimination and is a major challenge in improving the therapeutic index of liposomes for tumors. In this study, TMs was coated with PEG to avoid the MPS and prolong circulation time [20] and an anti-CD90 monoclonal antibody (MAb) was conjugated to TMs. The regression equation between the absorbance values and the concentration of anti-CD90 was A=18.89C-0.66. A and C are the absorbance values and the concentration of anti-CD90, respectively. The regression equation of the phospholipids was Y=16.83X+0.22. Y and X are the absorbance values and the concentration of phospholipids, respectively. The coupling efficiency of anti-human CD90 was 60.33%±5.78, corresponding to approximate 8 antibody molecules per liposome. Fe_3_O_4_ incorporated in the targeted TMs can be visualized by transmission electron microscope(TEM) (Figure 1A). Fe_3_O_4_ was clustered with a diameter of 10­­­-20 nm. Lipids layer of CD90@TMs was visible in correlative TEM image [21]. The average particle size in water was 130±4.6 nm (Figure 1B) and zeta potentials were negative (Figure 1C). The combination of anti-human CD90 to maleimide-1,2-distearoyl-sn-glycero-3-phosphoethanolamine-N-[methoxy(polyethylene glycol)-2000] (Mal-PEG_2000_-DSPE) was detected by fourier transform infrared spectroscopy (FTIR) (Figure 1D). The spectrum of Mal-PEG_2000_-DSPE showed weak C = O peak between 3600 cm^−1^ and 3200 cm^−1^ and weak N-H in 1674 cm^−1^. However, both of the two peaks increased in the spectrum of CD90-PEG_2000_-DSPE, indicating the successful combination of CD90 to Mal-PEG_2000_-DSPE. In the slide agglutination assay, when anti-mouse CD90 was added to CD90@TMs, an agglutination reaction formed, while saline added to CD90@TMs resulted in uniform scattering and no agglutination reaction was seen in control TMs (Figure 1E). The result further showed that the successful combination of anti-human CD90 to TMs.

**Figure 2:**
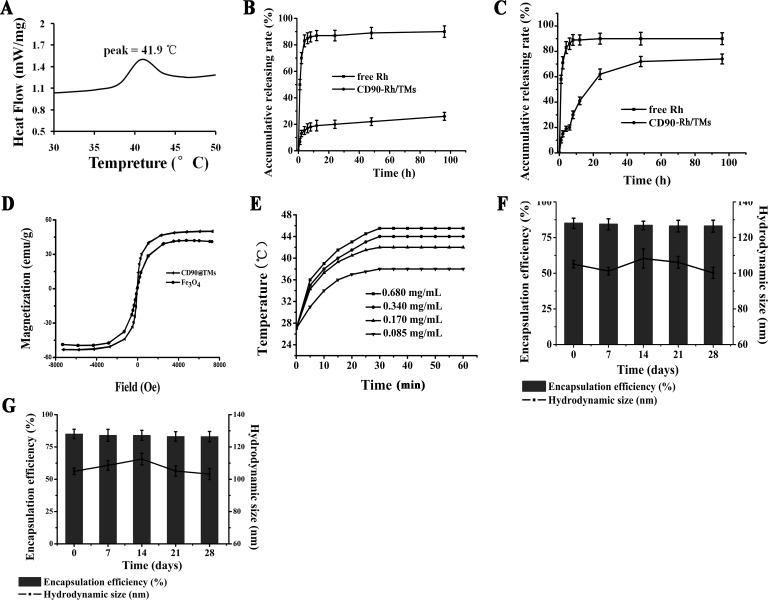
The characteristics of CD90@TMs **A.** Phase transition temperature of the CD90@TMs. **B.** Drug release property of Rh from the CD90-Rh/TMs and free Rh solution *in vitro* at 37±0.5°C (mean ± SD, *n* = 3). **C.** Release property of Rh from the CD90-Rh/TMs and free Rh solution *in vitro* at 42±0.5°C (mean ± SD, *n* = 3). **D.** Hysteresis Loops of Fe_3_O_4_ and CD90@TMs. **E**. Temperature increase curve of the CD90@TMs under an AMF (f = 200 kHz, I = 20 A). **F**. Physical stability of CD90-Rh/TMs in PBS at 4°C by the end of 1, 2, 3 and 4 weeks (mean ± SD, *n* = 3). **G.** Physical stability of CD90-Rh/TMs in DMEM/F12 medium at 4°C by the end of 1, 2, 3 and 4 weeks (mean ± SD, *n* = 3). Abbreviations: TMs, thermosensitive magnetoliposomes; CD90, cluster of differentiation 90; Rh, Lissamine rhodamine B; DSC, differential scanning calorimeter; AMF, alternating magnetic field; DMEM/F12, Dulbecco's Modified Eagle Media: Nutrient Mixture F-12.

When the temperature reaches the phase transition temperature, the lipid membrane of the thermosensitive liposomes is altered and the drugs in liposomes will leak out and diffuse into the target organ based on the concentration gradient. In contrast, unheated organs will have relatively low drug concentrations, which will reduce side effects. Based on this, in this study we used magnetic hyperthermia and thermosensitive liposomes to improve therapeutic effectiveness by accumulating drugs in the tumors. The phase transition temperature of CD90@TMs was evaluated by differential scanning calorimeter (DSC) (Figure 2A) and showed little change compared with pure DPPC (41.9 vs. 42°C). Temperature-sensitive release property *in vitro* was detected by the dynamic dialysis method at 37 ± 0.5°C and 41.9 ± 0.5°C. To evaluate the cumulative release rate, lissamine rhodamine B (Rh) was wrapped into the aqueous phase of the CD90@TMs to form CD90-Rh/TMs. The cumulative release rate of free Rh was five to seven-fold higher than CD90-Rh/TMs at 37±0.5°C after 1h (Figure 2B). However, the cumulative CD90-Rh/TMs release rate was < 30% after 120 h, which suggested that CD90-Rh/TMs was more stable at temperatures < the phase transition temperature. Meanwhile, the CD90-Rh/TMs showed a different release profile at a temperature near the phase transition temperature (Figure 2C). The cumulative release rate reached 50% after 24 h and 81% after 120 h. Thus, the as-synthesized DPPC-based liposomes showed good temperature-sensitive release property.

Specific absorption rate (SAR) is an important magnetic field parameter that induces heating and determines the magnetocaloric effect to the tumor in the presence of an AMF [22]. The SAR value of the CD90@TMs was 84±1.1 w/g. It was higher than pure Fe_3_O_4_ (67.2±1.2 w/g). This result was consistent with a previous report [23]. In addition, the iron content was 1.9 mg/mL. All of the hysteresis loops of the pure Fe_3_O_4_ and CD90@TMs shown in Figure 2D were superparamagnetic, which indicated that liposomes wrapped with Fe_3_O_4_ retained a superparamagnetic response. The results of thermodynamic test using different iron concentrations during AMF are shown in Figure 2E. The concentration of Fe_3_O_4_ inside the targeted liposomes was positively related to the temperature when the AMF intensity was fixed. During the initial 0.5 h, the sample showed a rapid temperature increase, which slowed after 25-30min. After 30 min, the temperature remained constant. 0.34 mg/mL Fe reached a temperature of 44°C and remained stable, which is within the effective treatment temperature range of 41-46°C.

To assess the stability of the targeted liposomes, the hydrodynamic size and encapsulation efficiency of CD90-Rh/TMs was determined in phosphate buffered saline (PBS, Figure 2F) or dulbecco's modified eagle media: nutrient mixture F-12 (DMEM/F-12, Figure 2G) medium. The stability of liposomes is an important consideration before a new efficient drug-loaded nano-system can be established and the change in mean particle size and encapsulation efficiency over time is a useful indicator of the stability of liposomal suspensions [24]. The hydrodynamic size of CD90-Rh/TMs and encapsulation efficiency of Rh showed no noticeable change over 4 weeks in two different solutions (Figure 2F and 2G), which indicated that the liposomes were stable and retained the encapsulated Rh when stored at 4°C.

### Analysis of cellular proliferation, differentiation, colony formation, migration and invasion and *in vivo* tumor formation

**Figure 3:**
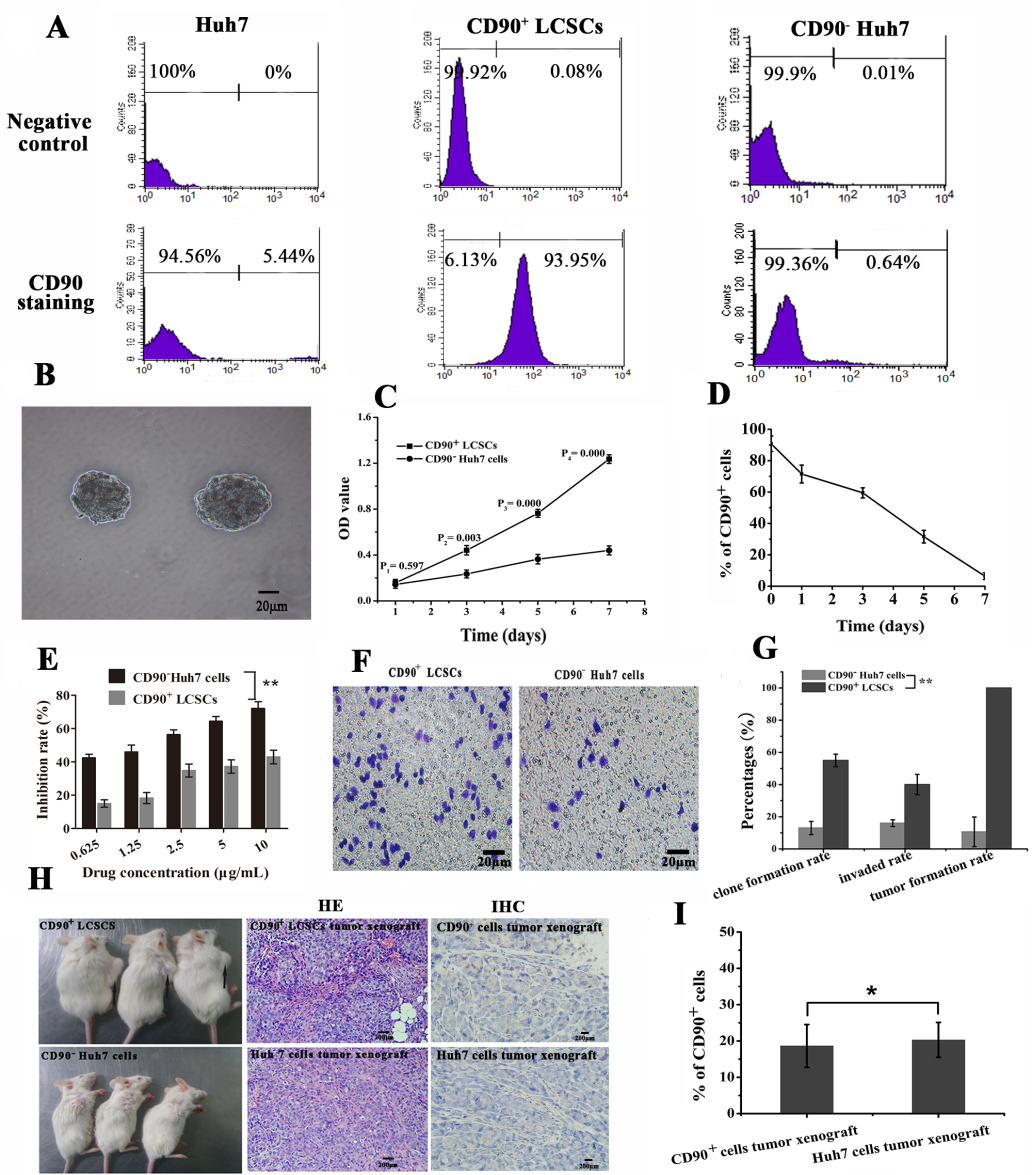
The stem characteristics of LCSCs **A.** The expression rates of CD90 in unsorted, sorted positive and sorted negative cells. **B.** Morphology of LCSCs spheroids after maintained in DMEM/F12 medium for 7 days (The bar = 20μm). **C.** Cell proliferation assay (mean ± SD, *n* = 3). **D.** Cell differentiation assay (mean ± SD, *n* = 3). **E.** Inhibition rate of CD90^+^ LCSCs and CD90^−^ Huh7 cells observed by CCK-8 assay (mean ± SD, *n* = 3). **F.** Invasion assay of CD90^+^ LCSCs and CD90^−^ Huh7 cells (The bar = 20μm). **G.** Clone formation rate, invaded rate and tumor formation rate (mean ± SD, *n* = 3). **H**. Tumorigenic images of NOD/SCID mice on two month after injection. (The xenografts tumors were marked by black arrow, the bar = 200μm). **I**. The percentages of CD90^+^ cells *in vivo.* **P < 0.05, *P > 0.05. Abbreviations: LCSCs, live cancer stem cells; DMEM/F12, Dulbecco's Modified Eagle Media: Nutrient Mixture F-12; CD90, cluster of differentiation 90; NOD/SCID, nonobese diabetic/severe combined immunodeficien.

CD90 can be used as a marker for a variety of stem cells, in addition to LCSCs and plays a role in the development of HCC [3, 4]. LCSCs can be enriched through fluorescence-activated cell sorting (FACS) and magnetic-activated cell sorting (MACS) using various cell surface markers. In this study, CD90^+^ LCSCs were isolated by MACS. The baseline expression of CD90 in whole populations of Huh7 cells *in vitro* was approximately 6.9±1.8% (Figure 3A). The purity of the sorted CD90^+^ cells and sorted CD90^−^ cells after magnetic separation was 90.8±4.9% and 0.7 ±0.6%, respectively (Figure 3A). The viability of the sorted CD90^+^ cells and sorted CD90^−^ cells after magnetic separation was about 99.1±1.9% and 98.9±1.2%. Spheroids formed during maintenance in DMEM/F12 medium for 7 days (Figure 3B). Cell proliferation assays indicated that the growth rate of CD90^+^ LCSCs was significantly higher compared to that of CD90^−^ Huh7 cells up to 7 days after cell sorting (*P* < 0.05, Figure 3C). It means that CD90^+^ LCSCs have a capacity for self-renewal. Flow cytometry was used to analyze the change of expression of CD90 in CD90^+^ LCSCs cultured with DMEM containing 10% FBS for 1 week. As shown in Figure 3D, the percentage of CD90^+^ cells dramatically decreased with culture time. After 1 week, the percentage of CD90^+^ cells had dropped to 6.2%, which was similar to the percentage in non-sorted Huh7 cells. The increase of OD value in CD90^+^ LCSCs group was due not to the fast proliferation of CD90^+^ cells but to an increase in the number of CD90^−^ cells. These results demonstrate that CD90^+^ LCSCs, which are a small and rare subset of Huh7 cells, have a capacity for self-renewal and differentiation to produce descendent CD90^−^ cells in culture. To determine the drug resistance of CD90^+^ LCSCs, the cells were treated with doxorubicin (DOX) at different concentrations, and the half maximal inhibitory concentration (IC50) values were calculated. Compared to CD90^−^ Huh7 cells, CD90^+^ LCSCs were more resistant to DOX (Figure 3E). The IC50 of CD90^+^ LCSCs and CD90^−^ Huh7 cells were 12.6±1.04 μg/mL and 1.07±0.13 μg/mL, respectively. Furthermore, CD90^+^ LCSCs exhibited higher ability of colony formation and invasion than CD90^−^ Huh7 cells (*P* < 0.05, Figure 3F, 3G). CD90^+^ cells sorted from liver cancer cell lines, liver cancer tissues or the peripheral blood of HCC patients displayed tumorigenic and metastatic capacity when injected into immunodeficient mice [3, 4, 25]. To determine whether the CD90^+^ LCSCs possessed a stem cell phenotype *in vivo*, xenograft tumors were induced by the subcutaneous injection of 2 × 10^4^ cells. The mice injected with CD90^+^ LCSCs formed tumors while CD90^−^ Huh7 cells formed no tumors after 3 weeks of observation. Two month after inoculation, all mice injected with CD90^+^ LCSCs developed tumors, whereas mouse inoculated with CD90^−^ Huh7 developed no tumors. The incidence of tumor xenografts in the CD90^+^ group was significantly higher than that in the CD90^−^ group (100% vs. 5.7±9.8%). Hematoxylin-eosin (HE) staining showed that tumor xenografts from implanted CD90^+^ LCSCs had histological features similar to Huh7 cells (Figure 3H). Immunohistochemical (IHC) staining showed that the expression rates of the CD90^+^ LCSCs transplantation group and the parent Huh7 established tumor group were 18.6±5.9% and 20.3±4.8%, respectively. It meant that CD90^+^ LCSCs could also produced CD90^−^ cells *in vivo*. These results showed that the CD90^+^ LCSCs isolated from Huh7 cells possessed the characteristics of CSCs.

### Targeting ability of CD90-Rh/TMs for CD90^+^ LCSCs *in vitro*

**Figure 4:**
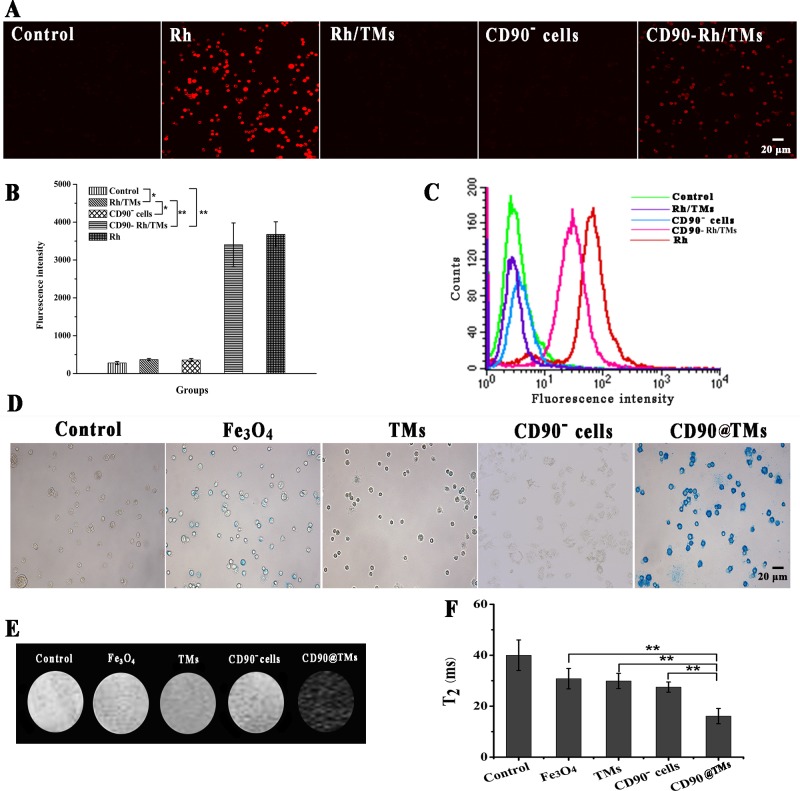
Specific targeting to CD90^**+**^ LCSCs with CD90-Rh/TMs **A.** Internalization of control group, CD90-Rh/TMs group, Rh group, Rh/TMs group and CD90^−^ Huh7 group detected by confocal microscopy, the bar = 20μm. **B.** The fluorescence intensity of Rh detected by confocal microscopy (mean ± SD, *n* = 3). **C.** Binding and internalization detected by flow cytometry of DMEM/F12 (control group), CD90-Rh/TMs (CD90- Rh/TMs group), free Rh(Rh group), Rh/TMs(Rh/TMs group) and CD90^−^ Huh7 cells incubated with CD90-Rh/TMs(CD90^−^ Huh7 group). **D.** Prussian blue staining of CD90^+^ LCSCs incubated with DMEM/F12 (control group), CD90@TMs (CD90@TMs group), pure Fe_3_O_4_ (Fe_3_O_4_ group), TMs (TMs group) and CD90^−^Huh7 cells incubated with CD90@TMs (CD90^−^ Huh7 group), the bar = 20μm. **E.** T_2_-weighted MRI images of control group, CD90@TMs group, Fe_3_O_4_ group, TMs group and CD90^−^ Huh7 group. **F.** T_2_ relaxation time of control group, CD90@TMs group, Fe_3_O_4_ group, TMs group and CD90^−^ Huh7 group (mean ± SD, *n* = 3). *P > 0.05; **P < 0.05. Abbreviations: Rh, Lissamine rhodamine; TMs, thermosensitive magnetoliposomes; DMEM/F12, Dulbecco's Modified Eagle Media: Nutrient Mixture F-12; CD90, cluster of differentiation 90.

To investigate whether the CD90-Rh/TMs could target CD90^+^ LCSCs *in vitro*, Rh encapsulated in those liposomes were incubated with cells and evaluated. Anti-CD19-targeted liposomes have been shown to be rapidly internalized into human B-lymphoma (Namalwa) cells within 60 min [26]. Using confocal microscopy, the fluorescence intensity of Rh (red) in CD90 targeted groups was higher than those of the control group and free Rh (Figure 4A, 4B), which was consistent with the flow cytometry results (Figure 4C). In flow cytometry results, uptake of CD90-Rh/TMs by LCSCs was higher than that of Rh/TMs and CD90^−^ cells but lower than Rh (Figure 4C). The uptake of CD90-Rh/TMs and Rh/TMs was lower than free Rh, which indicated that free Rh could cross the cell membrane and be efficiently enriched. However, these parameters were markedly different *in vivo*. Gabizon found that the removal rate of free doxorubicin was 450-fold higher than that of PEG-modified liposomes [27]. Owing to the biocompatible PEG modified on the membrane, liposomes hold over opsonization and consequently lead to comparatively longer blood circulation times, thus creating possibilities to target cancers when coated on target agents. These two results suggested that the CD90-Rh/TMs could be used to effectively deliver drugs into the cell.

To evaluate the capacity of the CD90@TMs to promote the capture of Fe_3_O_4_ by CD90^+^ LCSCs *in vitro*, cells were stained with Prussian blue and imaged by optical microscopy (Figure 4D). The targeted groups exhibited deep blue staining compared to those incubated with non-targeted liposomes, CD90^−^ cells or pure Fe_3_O_4_, indicating higher intracellular uptake of the Fe_3_O_4_ when modified with anti-CD90. Superparamagnetism Fe_3_O_4_ can reduce T_2_ relaxation time and show low signal intensity on T_2_-weighted MRI. To determine whether the targeted magnetoliposomes can be used as an effective molecular imaging labeling agent *via* MRI, CD90^+^ LCSCs were treated with CD90@TMs, TMs and pure Fe_3_O_4_ for 60 min. The results indicated that MRI signal intensity of the non-targeted group, pure Fe_3_O_4_ group and CD90^−^ cells group was sufficiently stronger than that of the targeted group (Figure 4E). The T_2_ relaxation time of the three groups was shown in Figure 4F. The T_2_ relaxation time of CD90^+^ LCSCs incubated with CD90@TMs was significantly lower than those of the cells incubated with non-targeted magnetoliposomes and pure Fe_3_O_4_ and the negative control cells and CD90^−^ cells (*p* < 0.05). These results suggest that targeted magnetoliposomes taken up by the CD90^+^ LCSCs low the MRI signal intensity of the cells on T_2_ weight sequence, thereby enabling effective MRI detection of cancer cells *in vitro*.

**Figure 5:**
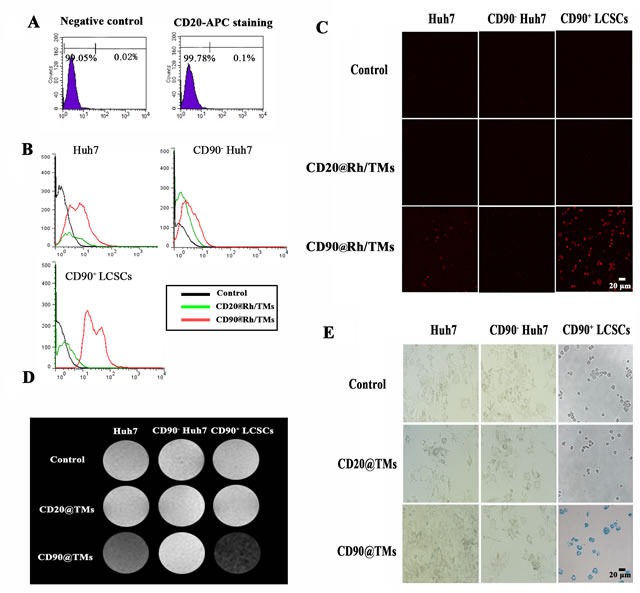
The targeting ability of TMs conjunct specific and non-specific antibody for CD90**+** LCSCs, CD90^**-**^ Huh7 and Huh7 **A.** The expression level of CD20 in Huh7. **B.** Binding and internalization of Rh detected by flow cytometry. **C.** Internalization of Rh detected by confocal microscopy, the bar = 20μm. **D.** T_2_-weighted MRI images of CD90^+^ LCSCs, CD90^−^ Huh7 and Huh7. **E.** Prussian blue staining of CD90^+^ LCSCs, CD90^−^ Huh7 and Huh7, the bar = 20μm. Abbreviations: Rh, Lissamine rhodamine; TMs, thermosensitive magnetoliposomes; DMEM/F12, Dulbecco's Modified Eagle Media: Nutrient Mixture F-12; CD90, cluster of differentiation 90.

The B-cell antigen CD20 is expressed on normal B cells and nearly all B-cell lymphomas. As it was shown in Figure 5A, there was nearly no expression of CD20 on the surface of Huh7. Hence, anti-CD20 MAb conjunct TMs (CD20@TMs) was chosen as a non-specific target group or irrelevant antibody target group to detect the specific cellular uptake of the CD90@TMs. The uptake with CD90-Rh/TMs and CD20-Rh/TMs using sorted positive, sorted negative and unsorted cells was observed by confocal microscopy. The fluorescence intensity of Rh in CD90^+^ cells group cultured with CD90-Rh/TMs was higher than that of the other group (Figure 5B and Table 1), which was consistent with the flow cytometry results (Figure 5C). The fluorescence intensity in Huh7 cells group cultured with CD90-Rh/TMs was slightly higher than that in the control group. The reason may be that there are still about 6% CD90^+^ LCSCs in the Huh7 cells. The difference among the control group, sorted positive, sorted negative and unsorted cells cultured with CD20-Rh/TMs had not statistical significance (*P* > 0.05). The capture of Fe_3_O_4_ by CD90^+^ LCSCs *in vitro* was detected by MRI (Figure 5D and Table 2) and Prussian blue staining (Figure 5E). The results showed that the content of Fe_3_O_4_ was highest in CD90^+^ LCSCs cultured with CD90@TMs compared to other groups. It suggested that CD90@TMs was a good target carrier for the delivery of the drug to CD90^+^ LCSCs.

**Table 1:** The fluorescence intensity of sorted positive, sorted negative and unsorted cells cultured with CD90 conjunct Rh/TMs and non-specific antibody conjunct Rh/TMs detected by confocal microscopy

	Huh7	CD90^−^ Huh7	CD90^+^ LCSCs
	$\left(\bar{x}\pm \text{S},\;n=3\right)$	$\left(\bar{x}\pm \text{S},\;n=3\right)$	$\left(\bar{x}\pm \text{S},\;n=3\right)$
Control	326.1±27.2	342.4±31.5	360.1±34.1
CD20@TMs	332.4±26.5	337.5±53.6	315.6±30.3
CD90@TMs	664.7±34.1	324.5±33.2	3589.5±47.2**

**Notes:** Data are presented as means ± standard deviation. Each experiment was repeated at least three times.

** Comparisons with the control group of CD90^+^ LCSCs, *P* < 0.05.

**Table 2:** T_2_ relaxation time(ms) of sorted positive, sorted negative and unsorted cells cultured with CD90 conjunct TMs and non-specific antibody conjunct TMs

	Huh7	CD90^−^ Huh7	CD90^+^ LCSCs
	$\left(\bar{x}\pm \text{S},\;n=3\right)$	$\left(\bar{x}\pm \text{S},\;n=3\right)$	$\left(\bar{x}\pm \text{S},\;n=3\right)$
Control	66.4±5.8	59.6±4.7	60.4±4.9
CD20@TMs	61.3±3.9	68.3±3.6	62.4±6.6
CD90@TMs	40.6±4.9	59.5±6.8	18.9±5.6**

**Notes:** Data are presented as means ± standard deviation. Each experiment was repeated at least three times.

** Comparisons with the control group of CD90^+^ LCSCs, *P* < 0.05.

**Figure 6:**
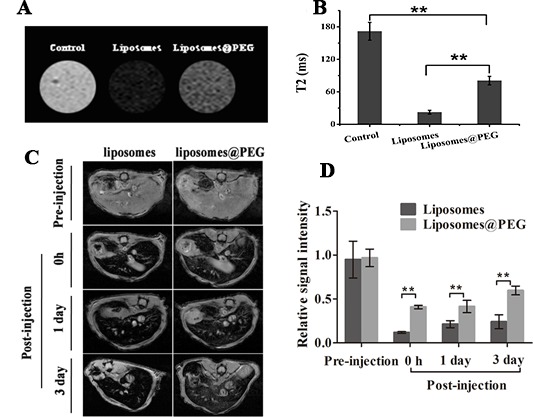
Non-specific uptake experiments *in vitro* and *in vivo* **A.** Non-specific uptake assay by RAW264.7 cells *in vitro*. **B.** T_2_ value of the RAW264.7 cells (mean ± SD, *n* = 3). **C.** Non-specific uptake assay by liver *in vivo*. **D.** Relative signal intensity of the liver. The T_2_ values were calculated and recorded as the mean ± standard deviation (*n* = 3). **P < 0.05.

It is worth noting that although in this experiment the CD90@TMs *in vitro* can bind to the CD90^+^ stem cells, whether it will be swallowed by macrophages reside in the reticuloendothelial system (RES) of liver is yet to know. We further performed the phagocytosis experiments to confirm whether the liposome is taken up by macrophages *in vitro*, and by the liver *in vivo*. It is straightforward to acquire T_2_ maps in non-tumor bearing mice prior to and following liposome administration. As shown in Figure 6A, liposomes group displayed lower T_2_WI signal intensity than liposomes@PEG after co-incubated with the same concentration of Fe_3_O_4_. The T_2_ relaxation time (Figure 6B) between liposome group and liposomes@PEG group showed significant statistically difference (*P* < 0.05), which demonstrated that PEG-modified liposomes could effectively reduce the phagocytosis of macrophages. *In vivo* MRI showed that T_2_WI (Figure 6C) intensity of liver tissue decreased obviously at different time points both in liposomes group and liposomes@PEG group. However, the relative signal intensity (Figure 6D) of liposomes group was significantly lower than liposomes@PEG group after injection (*P* < 0.05). Starting from 0 h, the T_2_ relaxation time at different time points had statistical difference between liposomes group and liposomes@PEG group(*P* < 0.05). This indicated that PEG-modified liposomes could decrease the non-specific uptake of macrophage in liver tissue remarkably, increase the time length of *in vivo* cycle of liposome and thus, increase the chance to combine with target cells. This provided powerful basis for *in vivo* imaging and therapy.

When the micro particle carriers of the drug were delivered into the systemic circulation, they were easily engulfed by the macrophage in RES [28]. Most of macrophages reside in the blood-rich RES system such as liver, lung, spleen and lymph nodes which are the targets of passive targeting drugs. However, once the drug does not target on RES system, phagocytosis of RES system would interfere with the effect of drug targeting. Therefore, avoiding phagocytosis of RES system has become a key subject in drug targeting. Liposome is a kind of lipid vesicle with the structure of phospholipid bilayer, and the aqueous phase core inside it can entrap many kinds of chemical drugs [29]. Because their membrane component has good compatibility with living tissue, liposome has currently been widely applied in medical field as a kind of drug delivery system. With PEG, a hydrophilic polymeric substance, modifying the surface of liposome, the opsonization of plasma components can be blocked, which markedly decreases the RES system, for example in liver, spleen and so on, to engulf liposome.

### The sensitivity of CD90^+^ LCSCs, CD90^−^ Huh7 cells and Huh7 cells to magnetic hyperthermia

**Figure 7:**
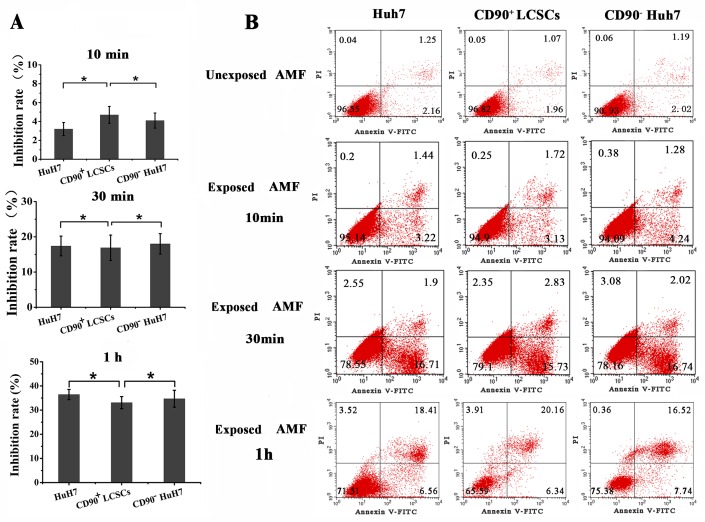
The sensitivity of CD90^**+**^ LCSCs, CD90^**-**^ Huh7 cells and Huh 7 cells to magenatic hyperthermia **A.** The sensitivity of CD90^+^ LCSCs, CD90^−^ Huh7 cells and Huh 7 cells to magenatic hyperthermia observed by MTT assay (mean ± SD, *n* = 3). **B.** The sensitivity of CD90^+^ LCSCs, CD90^−^ Huh7 cells and Huh 7 cells to magenatic hyperthermia observed by annexin-V- FITC/PI affinity assays. Abbreviations: TMs, thermosensitive magnetoliposomes; LCSCs, live cancer stem cells; mAb, monoclonal antibody; PI, propidium iodide; FITC, fluorescein isothiocyanate isomer I; MTT, 3-(4,5)-dimethylthiahiazo (-z-y1)-3,5-di- phenytetrazoliumromide; CD90, cluster of differentiation 90; TEM, transmission electron microscope.

Magnetic fluid hyperthermia is an effective procedure for tumor therapy. It is invariably induced by an AMF induction-heating device. Previous studies of magnetic fluid hyperthermia showed that the therapeutic effects could be strengthened by combination with radiotherapy and chemotherapy [30]. Magnetic fluid hyperthermia has been used to treat various cancers, such as breast cancer and liver cancer, and the effects have been well documented [31, 32]. However, the effect of magnetic fluid hyperthermia on LCSCs is unknown. The purpose of this study was to evaluate the sensitivities of CD90^+^ LCSCs, CD90^−^ Huh7 cells and Huh7 cells to TMs-mediated magnetic hyperthermia *in vitro*. The cells were heated using TMs to a predefined temperature (44°C) in an AMF for 10min, 30min and 1h. Changes in viability according to heating time were determined. As shown in Figure 7A, CD90^+^ LCSCs, Huh7 cells and CD90^−^ Huh7 cells exhibited identical sensitivities to magnetic fluid hyperthermia. The inhibition rate of CD90^+^ LCSCs after heating for 10min was 3.2±0.7% and there was no significant difference among CD90^+^ LCSCs, Huh7 and CD90^−^ cells group (*P* > 0.05). There was also no significant difference among the three groups when the heating time was 30min and 1h (*P* > 0.05). The apoptosis rate of Huh7, CD90^+^ LCSCs and CD90^−^ Huh7 cells was 3.1±0.6%, 4.4±0.6% and 4.7±0.5% when heating for 10min, 18.3±3.8%, 17.1±2.7% and 18.2±2.8% when heating for 30min, 23.8±3.2% 24.8±4.9% and 25.9±3.6% when heating for 1h, respectively. (Figure 7B and Table 3). There was no difference existed among CD90^+^ LCSCs, CD90^−^ Huh7 cells and Huh7 cells group. These results indicated that, unlike chemotherapy and radiotherapy, CD90^+^ LCSCs showed identical sensitivity to magnetic fluid hyperthermia compared with CD90^−^ Huh7 cells and Huh7 cells. This finding is consistent with a previous report [33]. Meanwhile, our data suggested that TMs-mediated magnetic hyperthermia can effectively reduce the number of CD90^+^ LCSCs. The result also indirectly suggests that magnetic hyperthermia is an effective therapy for patients resistant to chemotherapy or those unable to undergo radiotherapy for liver cancer.

**Table 3:** Apoptosis rate (%) of the Huh7 cells, CD90^+^ LCSCs and CD90^−^ Huh7 cells

	Control $\left(\bar{x}\pm \text{S},\;n=3\right)$	10min $\left(\bar{x}\pm \text{S},\;n=3\right)$	30min $\left(\bar{x}\pm \text{S},\;n=3\right)$	1h $\left(\bar{x}\pm \text{S},\;n=3\right)$
Huh7	3.8±2.8	3.1±0.6*	18.3±3.8*	23.8±3.2*
CD90^+^LCSCs	4.4±3.1	4.4±0.6*	17.1±2.7*	24.8±4.9*
CD90^−^Huh7	2.6±2.4	4.7±0.5*	18.2±2.8*	25.9±3.6*

**Notes:** Data are presented as means ± standard deviation. Each experiment was repeated at least three times.

* Comparisons between the three groups in each time, *P* > 0.05

### Targeted hyperthermia using CD90@TMs for CD90^+^ LCSCs *in vitro*

**Figure 8:**
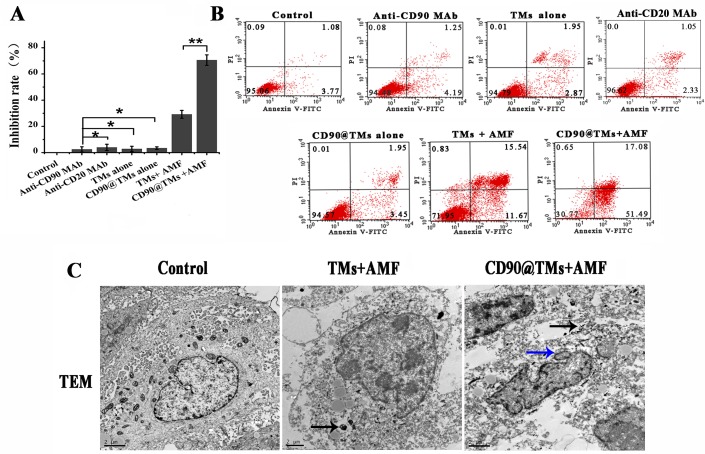
Targeted hyperthermia using CD90@TMs for CD90^**+**^ LCSCs **A.** Surviving rate of the CD90^+^ LCSCs observed by MTT assay (mean ± SD, *n* = 3). **B.** Apoptosis of the CD90^+^ LCSCs. *P > 0.05; **P < 0.05. **C.** Cell ultrastructure changes were observed by TEM. (The black arrows stand for the magnetic nanomaterial; the blue arrows stand for the nuclear morphology of cell apoptosis; the bar = 2μm). Abbreviations: TMs, thermosensitive magnetoliposomes; LCSCs, live cancer stem cells; mAb, monoclonal antibody; PI, propidium iodide; FITC, fluorescein isothiocyanate isomer I; MTT, 3-(4,5)-dimethylthiahiazo (-z-y1)-3,5-di- phenytetrazoliumromide; CD90, cluster of differentiation 90; TEM, transmission electron microscope.

Magnetic particle can decrease cell viability by targeted intracellular hyperthermia or by some other mechanism that is SAR/field strength dependent [34]. In assays of hyperthermia sensitivity, TMs-mediated hyperthermia treated CD90^+^ LCSCs resulted in a inhibition rate of 33.1±2.3%. We next investigated whether the targeted magnetoliposomes, CD90@TMs, could kill CD90^+^ LCSCs more effectively than TMs. As shown in Figure 8A, the inhibition rate of the targeted hyperthermia group using CD90@TMs was increased to 70.57±4.1%. However, non-targeted hyperthermia under identical conditions increased the inhibition rate to 29.21±3% while the anti-CD90, anti-CD20, TMs and CD90@TMs not exposure to AMF nearly showed no influence on the growth of the CD90^+^ LCSCs compared with control cells incubated with DMEM/F12. Thus, targeted hyperthermia was more effective in terms of inhibiting cell proliferation than non-targeted hyperthermia (*P* < 0.05).

A higher level of magnetic particle uptake by cell could contribute to an increase in intracellular hyperthermia leading to decrease cell viability [34]. To confirm that CD90@TMs were more effective in killing CD90^+^ LCSCs than non-target TMs, apoptosis was analyzed by flow cytometry (Figure 8B and Table 4). The proportion of apoptosis events in CD90^+^ LCSCs treated with CD90@TMs and TMs exposed to magnetic hyperthermia were 66.3±6.9% and 19.9±6.4%, respectively. Similar to the 3-(4, 5-dimethyl-2-thiazolyl)-2, 5-diphenyl-2-H-tetrazolium bromide (MTT) assay results, anti-CD90 targeted hyperthermia resulted in a 3.3-fold higher apoptosis rate compared to non-targeted hyperthermia (*P* < 0.05). The proportions of cells in apoptosis stage following incubation with DMEM, anti-CD90, anti-CD20, TMs and CD90@TMs were 4.1±1.1%, 4.5±2.4%, 3.8±2.9%, 5.2±1.4%, and 5.7±1.6%, respectively. This indicated that anti-CD90, anti-CD20, TMs and CD90@TMs alone were not toxic to CD90^+^ LCSCs.

**Table 4:** Apoptosis rate (%) of the CD90^+^ LCSCs

Group	Apoptosis rate $\left(\bar{x}\pm \text{S},\;n=3\right)$
Control	4.1±1.1
TMs alone	4.5±2.4
Anti-CD90 mAb	5.2±1.4
Anti-CD20 mAb	3.8±2.9
CD90@TMs alone	5.7±1.6
TMs+AMF	19.9±6.4**
CD90@TMs+AMF	66.3±6.9**

**Notes:** Data are presented as means ± standard deviation. Each experiment was repeated at least three times.

** Comparisons between the experimental groups and the control group, *P* < 0.05.

Cell ultrastructure changes and the distribution of the Fe_3_O_4_ nanoparticles at the cellular level were observed by TEM. Because the groups of anti-CD90, TMs and CD90@TMs alone showed no toxic to CD90^+^ LCSCs, we only observed the distribution of the Fe_3_O_4_ and cell changes in the group of control, TM+AMF and CD90@TMs+AMF by TEM. Figure 8C gave an impression of the particle uptake into the CD90^+^ LCSCs in TMs+AMF and CD90@TMs+AMF groups (black arrows). The nuclear membrane in the control group was clear with intact nuclei. In contrast, in hyperthermia group, the nucleus became deformative and the nuclear membrane fractured. The typical morphological changes of apoptosis included chromatin condensation and aggregation at the periphery of the nucleons could be seen in the CD90@TMs+AMF group (blue arrow). The results mean that hyperthermia can killed CD90^+^ LCSCs. The effect of target hyperthermia is superior to non-targeted hyperthermia.

**Figure 9:**
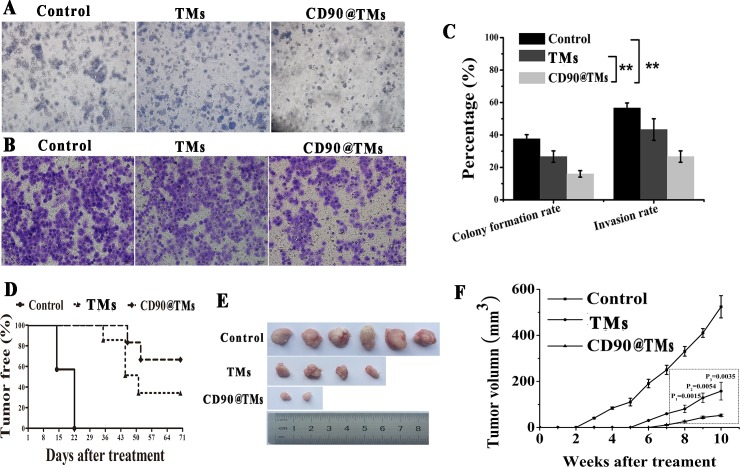
Effects of hyperthermia on the CD90^**+**^ LCSCs characteristic **A.** Effect of different treatment methods on colony formation of CD90^+^ LCSCs (The bar = 50μm). **B.** Effect of different treatment methods on invasion of CD90^+^ LCSCs (The bar = 20μm). **C.** Effect of different treatment methods on colony formation, invasion of CD90^+^ LCSCs (mean ± SD, *n* = 3). **D.** Effect of different treatment methods on tumorigenic ability (*n* = 6). Kaplane-Meier plot showed that the oncogenicity of mice in the control group is significantly higher than that in TMs and CD90@TMs group (*p* < 0.05). **E.** Tumors morphology in different treatment methods. **F**. Effect of different treatment methods on tumor growth of CD90^+^ LCSCs (mean ± SD, *n* = 6). **P < 0.05. Abbreviations: TMs, thermosensitive magnetoliposomes; LCSCs, live cancer stem cells; CD90, cluster of differentiation 90; AMF, alternating magnetic field.

There is no established method of determining the effects of hyperthermia on CSCs. Therefore, we investigated the characteristics of CSCs, including drug resistance, colony formation and invasion, as well as tumorigenic ability. The MTT and apoptosis assays showed that anti-CD90, anti-CD20, TMs alone and CD90@TMs alone exhibited no toxicity against CD90^+^ LCSCs. So, CD90@TMs and TMs mediated hyperthermia were chosen to detect the effect of target hyperthermia and non-target hyperthermia to drug resistance, colony formation invasion and tumor formation. CD90^+^ LCSCs derived from Huh7 cells treated with CD90@TMs mediated hyperthermia showed a marked decrease in colony formation rate and invasion rate compared to TMs-mediated hyperthermia (Figure 9A, 9B, 9C; *P* < 0.05). IC50 of CD90@TMs (3.7±0.2μg/mL) group was significant (*P* < 0.05) smaller than control group (15.7±3 μg/mL) and TMs group (7.8±1.1μg/mL). Magnetic hyperthermia exposure inhibited the tumorigenic ability of CD90^+^ LCSCs, as 27.3±9.8% of the mice treated with TMs mediated hyperthermia and 78±19.1% treated with CD90@TMs mediated hyperthermia exhibited no tumors at 70 days after the injection (Figure 9D, 9E). Compared with TMs-mediated hyperthermia, CD90@TMs mediated hyperthermia showed a marked delay in tumor generation and decreased tumorigenesis and tumor volume (Figure 9D, 9F). This suggested that the CD90@TMs-mediated intracellular magnetic hyperthermia effectively reduced or, in some cases, eliminated scarce CSCs.

### Effect of targeted hyperthermia on CD90^+^ LCSCs-bearing mice

**Figure 10:**
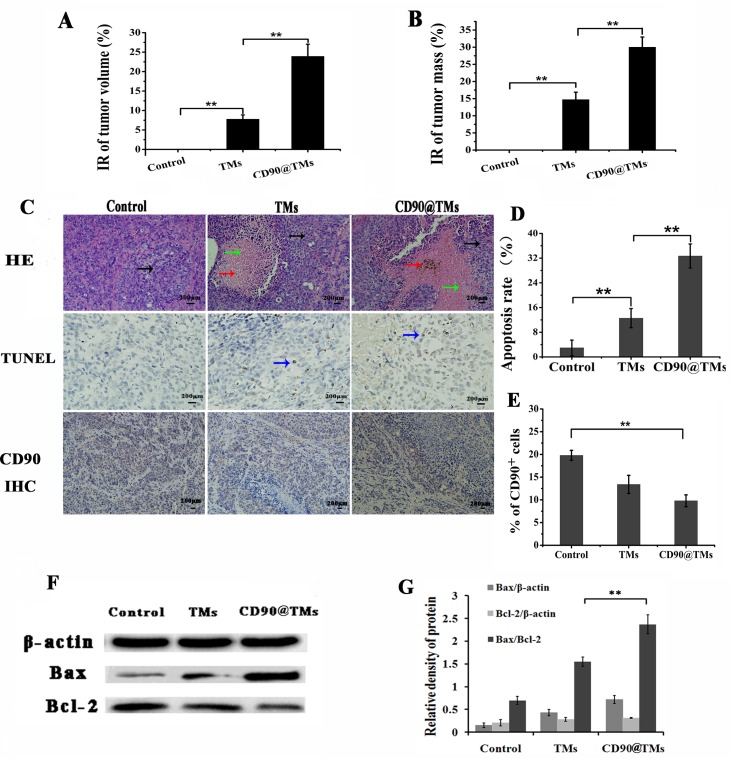
Effect of targeted hyperthermia on CD90^**+**^ LCSCs bearing mice **A.** Inhibition rate of tumor volume in different experimental groups (mean ± SD, *n* = 6). **B.** Inhibition rate of tumor mass in different experimental groups (mean ± SD, *n* = 6). **C.** Tumor tissues of CD90^+^ LCSCs-bearing mice stained by HE staining, TUNEL staining and CD90 IHC staining assay (the black arrows stand for the tumor tissues; the green arrows stand for the necrosis and collapse of tumor cells; the red arrows stand for the magnetic nanomaterial, the blue arrows stand for the typical apoptosis characteristics), the bar = 200μ m. **D.** Apoptotic cells in tumor tissues of CD90^+^ LCSCs-bearing mice (mean ± SD, *n* = 6). To assess the fraction of apoptotic cells, the count of TUNEL-positive cells was calculated from five sections. **P < 0.05. **E.** CD90^+^ cells in tumor tissues after being treated. **P < 0.05. **F**. Bax and Bcl-2 analysis by western blot. **G**. Relative density of Bax and Bcl-2. **P < 0.05. Abbreviations: IR, inhibition rate; TMs, thermosensitive magnetoliposomes; CD90, cluster of differentiation 90; HE, haematoxylin-eosin; TUNEL, terminal deoxynucleotidyl transferase-mediated dUTP-biotin nick end labeling; IHC, immunohistochemical.

Hyperthermia is a new and effective cancer treatment method that has gained popularity in recent years [35]. The ability of CD90@TMs to work *in vivo* was assessed in CD90^+^ LCSCs-bearing mice. The effect was assessed by determining tumor volume and mass over a treatment period of 7 days, and by HE staining at the end of the treatment period. The mice were divided into three groups: control group; TMs group and CD90@TMs group. As shown in Figure 10A, the CD90@TMs group had the most significant reduction in tumor size compared to control (*P* < 0.05). The inhibition rate of tumor volume increased to 23.92±3.12% in the CD90@TMs group and more than 2-fold in the TMs group. The tumor mass inhibit rate of the CD90@ TMs group was higher than those in the control group and TMs group (Figure 10B). The inhibition rates of tumor mass of the TMs group and CD90@TMs group were 14.7±2.2% and 30.0±2.9%, respectively. On day 7, all mice were sacrificed. HE staining was performed. As shown in Figure 10C, visible Fe_3_O_4_ sediment (red arrows) was found in the tumor tissues (black arrows) taken from thermotherapy groups and these sediments were surrounded by necrotic tumor cells (green arrows). The most severe damage occurred in the CD90@TMs group, which may explain why the highest decrease in tumor volume occurred in this group. To identify the cell apoptosis rate *in vivo*, terminal deoxynucleotidyl transferase-mediated dUTP-biotin nick end labeling (TUNEL) staining (Figure 10C, 10D) was performed. The largest number of TUNEL positive cells was found in the tumor tissues of the CD90@TMs group. A significant difference was detected between the CD90@TMs group and the other groups (*P* < 0.05). Compared with TMs group, CD90 expression decreased more significantly in CD90@TMs group (*P* < 0.05, Figure 10C, 10E). Research on hyperthermia is lagging and further study of the underlying molecular mechanism is needed. It is known that apoptosis contributes to hyperthermia-induced tumor killing alone or when combined with other therapies [36]. Initiation of apoptosis directly regulates cell fate decisions and the ratio of Bcl-2 family members is a crucial modulating factor in the process [37]. For example, the ratio of Bax/Bcl-2 is a trigger of apoptosis [38]. Studies have shown that hyperthermia can induce apoptosis through the mitochondrial pathway, although how hyperthermia activates mitochondria remains unknown [39]. The ratio of Bax and Bcl-2 may play an important role in this regard [40], which was supported by Western blot results demonstrating that hyperthermia led to a down-regulation of the apoptosis suppression protein Bcl-2 and up-regulation of Bax compared with the control group (*P* < 0.05, Figure 10F, 10G). This suggested that hyperthermia could induce apoptosis by regulating the expression of the apoptosis-related proteins Bcl-2 and Bax during CSC-targeted therapy because the anti-CD90 targeted hyperthermia group showed greater down-regulation of Bcl-2 and an up-regulation of Bax compared to the TM-mediated magnetic hyperthermia group (*P* < 0.05). Above all, CD90@TMs is more effective in eradicating CD90^+^ LCSCs compared to TMs *in vivo*.

The isolation of CSCs based on stem cell surface markers provides an important tool for investigating the properties of LCSCs and has represents a potential molecular target for LCSC therapy [41]. Thymocytes, T-cells, fibroblasts, endothelial cells, neurons and LCSCs share common cell surface markers, which could result in damage to these cells if CD90 was used as a molecular target. However, magnetic fluid hyperthermia has the advantage of being able to precisely heat the tumor tissue. When Fe_3_O_4_ particles reach the tumor, a magnetic field induction-heating device is applied to heat the magnetic fluid taken up by the CD90^+^ LCSCs and minimize damage to normal tissues. Several studies have indicated that CSCs are resistant to conventional chemotherapy and radiotherapy. However, when such traditional therapies are combined with hyperthermia, this resistance could be reduced [42] or eliminated [43]. Furthermore, effective elimination of CSCs through hyperthermia has been reported by Sadhukha [13]. Thus, hyperthermia may be useful for the treatment of HCC through purging LCSCs.

These results describe the first use of hyperthermia to target and kill LCSCs. Our findings demonstrate that the CD90@TMs can effectively target LCSCs and show increased tumor cell killing efficacy compared with TMs *in vitro* and *in vivo*. This indicates that hyperthermia may be a promising modality for the control of human tumors.

## MATERIALS AND METHODS

### Cells and animals

Huh7 cells were purchased from the Institute of Biochemistry and Cell Biology (Shanghai Institute of Biological Sciences, Chinese Academy of Sciences). Cells were cultured in RPMI 1640 medium (Gibco, Grand Island, NY, USA) supplemented with 10% fetal bovine serum (FBS, Shanghai, China), 100 U/mL penicillin, and 100 mg/mL streptomycin and maintained at 37°C with 5% CO_2_ in a humidified incubator. CD90^+^ LCSCs were isolated from Huh7 cell lines through MACS and cultured with DMEM/F12 (1:1) (Gibco, NY, USA) containing 20 μg/L recombinant human epidermal growth factor (hEGF), 20 μg/L basic fibroblast growth factor (bFGF) and 2% B27 in a low-adhesion culture bottle.

Nonobese diabetic/severe combined immunodeficien mice (NOD/SCID mice, male, 5 weeks old) were purchased from the vital river experimental animal technical co., LTD in Beijing, China. All the mice were maintained in the Sterile Barrier System of Medical School, Southeast University, China. All experiments involving animals were performed in compliance with the guidelines of the Animal Care Committee of Southeast University, Nanjing, China. All animals received humane care in compliance with the Principles of Laboratory Animal Care formulated by the National Society for Medical Research and the Guide for the Care and Use of Laboratory Animals prepared by the Institute of Laboratory Animal Resources and published by the National Institutes of Health (NIH Publication No. 86-23, revised 1996).

### Materials

A MidiMACS starting kit was purchased from Miltenyi Biotec (Germany). Purified mouse anti-human CD90 was purchased from BD Pharmingen. Rh was purchased from Aladdin (Shanghai, China). DAPI Fluoromount-G^®^ was purchased from Southern Biotech (Birmingham, USA). DPPC, PEG_2000_-DSPE, and MAL-PEG_2000_-DSPE were purchased from Lipoid (Germany). Dihydro-2(3H)-thiophenimine hydrochloride (Traut's reagent) was purchased from Sigma-Aldrich (St. Louis, USA). All solvents were of high-performance liquid chromatography grade.

### Preparation of CD90@TMs

Fe_3_O_4_ was prepared as described by Li using chemical co-precipitation [44]. 56mg of DPPC, 6mg of Chol and 1mg of PEG_2000_-DSPE was dissolved in 6mL of methanol and 3mL of chloroform (v/v, 2:1) [45]. The lipids formed a homogeneous thin film following vacuum rotary evaporation at 42°C. 10 mL of PBS buffer (0.1 M, pH = 7.4) mixed with Fe_3_O_4_ (2mg/mL) was added for 1 h to hydrate the solution and obtain TMs. 10 mL of PBS buffer (0.1 M, pH = 7.4) mixed with 10 mg Rh as a red fluorescent marker and 20 mg Fe_3_O_4_ was added for an additional 1 h to obtained Rh/TMs. The liposomes were placed in a dialysis bag (1000 Da) which was immersed in PBS buffer (0.1 M, pH = 7.4) to remove non-encapsulated Rh. Finally, the non-encapsulated Fe_3_O_4_ was removed by centrifuge at 1500 rpm for 20 min three times.

CD90@TMs were prepared as described previously using post-insertion [46, 47] with a slight modification. In brief, sulfhydryl was introduced to purified mouse anti-human CD90 (1mg) by incubating with 0.09 mg of traut's reagent in 0.5 mL PBS buffer (0.1 M, pH = 8.0). Anti-human CD90-SH was incubated with liposomes of 4 mg of Mal-PEG_2000_-DSPE and 13.6 mg of PEG_2000_-DSPE(the initial molar ratio of PEG _2000_ -DSPE relative to Mal-PEG_2000_-DSPE was 4:1) at 4°C overnight in 4 mL of PBS (0.1 M, pH = 8.0). Non-conjugated Abs was removed by centrifuge at 12000 g for 20 min three times. Anti-human CD90-PEG_2000_-DSPE was then incubated with 36 mg of TMs (DPPC) (the initial molar ratio of PEG_2000_ -DSPE relative to DPPC was 0.05:1) for 60 min at 42°C in PBS buffer (0.1 M, pH = 8.0).

The bicinchoninic acid (BCA) assay (Micro BCA Protein Assay Kit, Pierce Biotechnology, USA) was used to detect the Abs content according to the manufacturer's instructions as described by Zhang et [47]. The CD90 absorbance of the diluted protein obtained from the albumin (BSA) standard solution(0, 0.025, 0.05, 0.1, 0.2, 0.4 μg protein/μL) was used for standard curve preparation by using a spectrophotometer (Bio-Tek, Winooski, USA) at 568 nm. The phospholipid concentration of liposome was determined using the Stewart method [48]. A 1-mL aliquot of CD90 liposome was dispersed into 1 mL chloroform solution and then was added into 2.0 mL ammonium ferrothiocyanate solution (3.04g ammonium thiocyanate, 2.7g ferric chloride, 100mL dH_2_O). After mixing for 1 min and centrifuging for 10 min at 2000 rpm, the supernatant was analyzed by using ultraviolet-visible spectrophotometry (UV-VIS spectrophotometry, UV-3600, Shimadzu, Tokyo, Japan) for the phospholipid concentration at 488 nm. The spectrophotometrical analyzation of liposome based on the principle that phospholipids generate stable color complexes (488 nm) with ammonium ferrothiocyanate in the organic solution at room temperature. The absorbance of the diluted sample obtained from DPPC standard solution was used for standard curve preparation. The coupling efficiency of anti-human CD90 was calculated. The following parameters were used to calculate the final anti-CD90 density on the resulting liposome (equation below): (a) the molecular mass of CD90 (150 kD); (b) the mass of CD90; (c) the molecular mass of DPPC (734 D); (d) the mass of the phospholipids; (e) the approximate number of phospholipid (90000) [49].

anti−CD90 moleculars per liposome=the mass of anti−CD90150000the mass of phospholipid734×90000

CD90-Rh/TMs was prepared similarly.

### Characterization of CD90@TMs

#### Morphology and size analysis

The morphology of Fe_3_O_4_ and CD90@TMs was assessed by TEM (Hitachi, Tokyo, Japan).

Liposome size and zeta potentials were determined using ZetaPlus analysis (Brookhaven Instruments Co., Holtsville, NY, USA).

#### Fourier translation infrared spectroscopy and slide agglutination assay

FTIR was used to evaluate the successful conjunction between CD90 and Mal-PEG_2000_-DSPE. The spectrum of Mal-PEG_2000_-DSPE and CD90-PEG_2000_-DSPE was detected, respectively. The slide agglutination method [50] was used to evaluate the combination of anti-human CD90 with TMs and the biological activity of CD90@TMs *in vitro*. First, CD90@TMs and TMs were added to two clean slides, respectively. Then the anti-mouse CD90 or saline were added in two TMs and the slides were incubated for 30 min at 37°C. The agglutinate reaction was observed by optical microscopy.

#### Characteristic analysis of thermal sensitivity

CD90@TMs was analyzed by differential thermal analysis to evaluate the phase transition temperature. To identify the release mechanism of TMs, Rh release from the nanoparticles in PBS (pH 7.4) at 37°C and 42°C was evaluated using the dynamic dialysis method. Rh concentration release from each sample at 0, 1, 2, 4, 6, 8, 12, 24, 48, 96 and 120 h was quantified using an F-2700 fluorescence spectrophotometer (HITACHI, Tokyo, Japan; ex/em: 570/600 nm).

#### Stability analysis

To assess the physical stability of the nanoparticles, the hydrodynamic size of CD90-Rh/TMs and encapsulation efficiency of the Rh in PBS and DMEM/F12 medium were analyzed within 24 h. The particle size and encapsulation efficiency were measured for 4 weeks with storage at 4°C with ambient humidity.

#### Hysteresis loops, specific absorption rate and increasing temperature tests

Iron content was measured using 1, 10-phenanthroline spectrophotometry as described by Wu [22]. The Fe_3_O_4_ powder and freeze-dried CD90@TMs were placed in a vibrating sample magnetometer (VSM) to determine the magnetic properties at room temperature. The CD90@TMs was placed on SPG-06A high-frequency induction heating equipment (Shenzhen, China) (f = 200 kHz, I = 20 A) for 60 min to do the thermodynamic test. The SAR values of Fe_3_O_4_ and CD90@TMs were calculated as described by Xie et al.[51].

### Isolation of CD90^+^ LCSCs

Huh7 cells were mixed and incubated with 20 μL CD90 MicroBeads (Miltenyi Biotec, Bergisch Gladbach, Germany) per 10^7^ total cells at 4°C for 30 min. The cells were then washed and were applied onto a MiniMACS Column, which was placed in the magnetic field. The magnetically labeled CD90^+^ Huh7 cells were absorbed by the magnetic field and retained in the MiniMACS column, while CD90^−^ Huh7 cells passed through the column and were collected. Finally, the MiniMACS column was removed from the magnetic field, the magnetically retained CD90^+^ cells, called CD90^+^ LCSCs were collected by firmly pushing the plunger into the column. The sorted CD90^+^ LCSCs were cultured with DMEM/F12 medium 4 h and then used in CSC characteristics analysis, targeting ability assay and treatment trial. The purity of the CD90^−^ Huh7 and CD90^+^ LCSCs were analyzed by Flow Cytometer (FCM, BD Biosciences, USA) after being stained with CD90-APC (Miltenyi Biotec, Bergisch Gladbach, Germany). Trypan blue staining was used as an index of cell activity.

### Analysis of the LCSC characteristics

#### Cellular proliferation and differentiation experiment

The CD90^+^ LCSCs and CD90^−^ Huh7 cells were seeded in 96-well plates at 1 × 10^3^ /well and cultured in complete RPMI l640 with 10% FBS for 1, 3, 5 and 7 days. Cell proliferation was assayed using a cell counting kit-8 (CCK8) assay. CD90^+^ LCSCs were cultured with DMEM medium supplemented with 10% fetal bovine serum. At 1, 3, 5, and 7 d, cells were collected and incubated with APC-conjugated anti-CD90 monoclonal antibody at 4°C for 30 min, and then analyzed by a Flow Cytometer (FCM, BD Biosciences, USA).

#### Drug resistance

Drug resistance of the cells to DOX was measured using a CCK8 assay. The rate of cell growth inhibition was calculated by the formula: Inhibition rate = (OD value of the control group - OD value of experimental group)/(OD value of the control group - OD value of blank Group). Drug doses to inhibit 50% of cell growth (IC50 values) were then calculated by modified Kou-type method:

lg IC50=Xm−I(P−(3−Pm−Pn)4)

Where Xm: lg Maximum dose; I: lg (maximum dose/adjacent dose); P: sum of positive response rate; Pm: the largest positive response rate; Pn: the smallest positive response rate.

#### Colony formation

The colony-formation capacity of CD90^+^ LCSCs and CD90^−^ Huh7 cells were detected by re-suspending cells in 2-mL cell culture medium contain 0.3% low melting temperature agarose at 1500 cells per well and then seeding in six-well plates covered with 0.6% agarose. The six-well plates were then incubated for 2 weeks at 37°C, 5% CO_2_ until colonies formed. Colonies >50 cells were counted as described previously [52].

#### Invasion assay

To analyze cell invasion, corning invasion chambers were coated with matrigel. The CD90^+^ LCSCs and CD90^−^ Huh7 cells were re-suspended in 200 μL of DMEM at 2.0 × 10^3^/ mL and were transferred to the lower chambers containing 500 μL of DMEM with 10% FBS. After 24 h, invasive cells were fixed in 4% paraformaldehyde for 30 min, stained with 0.1% crystal violet solution and counted under an inverted microscope.

#### Tumor formation experiment

NOD/SCID mice were randomized into the following three groups: control, CD90^+^ LCSCs and CD90^−^ Huh7 cells (*n* = 6 mice /group). Tumors were induced by subcutaneous injection of 2 × 10^4^ cells. The half numbers of the mice were observed two month and the others were euthanized when the tumors were 100 mm^3^. The tumors were excised, fixed in 10% formalin and embedded in paraffin. HE staining was used to observed morphological and histological changes. IHC staining was used to detect the expression of CD90 *in vivo*.

### Targeting ability of CD90@TMs for CD90^+^ LCSCs

#### Flow cytometry

The CD90^+^ LCSCs used in the targeting experiment after 4 h in culture after sorted by MACS. Rh was wrapped incorporated into TMs as a fluorescent indicator of CD90@TM targeting to CD90^+^ LCSCs. Non-specific antibody was conjunct with TMs (CD20@TMs) to test the specificity of cellular uptake of the CD90@TMs. The expression of CD20 was detected by flow cytometry after being stained with CD20-APC (Miltenyi Biotec, Bergisch Gladbach, Germany). Flow cytometry was used to investigate the binding of CD90-Rh/TMs to CD90^+^ LCSCs. CD90^+^LCSCs were incubated with CD90-Rh/TMs, Rh/TMs and Rh (5 μg/mL) for 1 h at 37°C in 5% CO_2_. CD90^+^ LCSCs incubated with medium or CD20-Rh/TMs and CD90^−^ Huh7 cells incubated with CD90-Rh/TMs for 1 h were used as controls.

#### Confocal laser scanning microscopy

Confocal laser scanning microscopy was used to assess the uptake and distribution of LCSCs to CD90-Rh/TMs. CD90^+^LCSCs were incubated with CD90-Rh/TMs, Rh/TMs and 5 μg/mL Rh for 1 h at 37°C in 5% CO_2_. CD90^+^ LCSCs incubated with medium or CD20-Rh/TMs, CD90^−^ Huh7 cells incubated with CD90-Rh/TMs for 1 h were used as controls.

#### MRI assay

The uptake of Fe_3_O_4_ was detected by 7.0-Telsa Micro MRI (Bruker, Germany). CD90^+^ LCSCs were seeded onto the 6-well plates at 1 × 10^4^ cells/well and incubated with CD90@TMs, TMs and pure Fe_3_O_4_ for 1 h at 37°C in 5% CO_2_. CD90^+^ LCSCs incubated with medium or CD20@TMs, CD90^−^ Huh7 cells incubated with CD90@TMs for 1 h was used as controls. Cells were resuspended by centrifugation at 1000 rpm for 5 min and the supernatant was aspirated completely. Cells were then washed with PBS for three times and resuspended in 1% agarose (0.5 mL) in the ependoff tube. The ependoff tubes were scanned with a 7.0-Tesla MRI system. A T_2_ mapping sequence (TR: 2000 ms, TE: 30 ms, matrix 256 × 256, field of view [FOV] 5 × 5cm, 1mm thick, 3.0cm diameter body coil) was used to evaluate transverse relaxation time. The T_2_ values were calculated and recorded as the mean ± standard deviation (*n* = 3).

#### Prussian blue staining

The cellular uptake of Fe_3_O_4_ was evaluated Prussian blue staining. After incubation with CD90@TMs, TMs and pure Fe_3_O_4_ for 1 h at 37°C in 5% CO_2_, the cells were stained with Prussian blue solution at 37°C for 30 min. CD90^+^ LCSCs incubated with medium or CD20@TMs, CD90^−^ Huh7 cells incubated with CD90@TMs for 1 h were used as controls.

#### Non-specific uptake assay *in vitro* and *in vivo*

In this study, we prepared the TMs coated with PEG to reduce the uptake of MPS and get longer circulation lifetimes. The experiments *in vitro* and *in vivo* were used to confirm that the TMs coated with PEG (called liposomes@PEG) could reduce the uptake by MPS compared to the TMs not modified with PEG (called liposomes).

RAW264.7 macrophages were seeded onto the 6-well plates at 1 × 10^4^ cells/well and incubated with liposomes@PEG and liposomes for 2 h at 37°C in 5% CO_2_. The value of T_2_ was detected on a 7.0-Tesla MRI system.

The liposomes@PEG and liposomes were injected in the health mice in the 5mg Fe per kg of the mice through tail vein, respectively. MRI of the liver was performed before injection and at 0h, 1d, and 3d post-injection. The mapping sequence for the liver was as follows: MSME-T_2_WI: FOV = 35 mm × 35 mm, TR = 3000 ms, TE = 20 ms, slice thickness = 0.8 mm, matrix = 256 × 256. FLASH-T_2_*sequence: FOV = 35 mm × 35 mm, TR = 408 ms, TE = 3.5 ms, slice thickness = 0.8 mm, matrix = 256 × 256. The relative signal intensity of the liver was calculated as followed: Relative signal intensity = T_2_ value of the liver/ T_2_ value of the muscle. T_2_ relaxation times were measured by manually drawing a region of interest (ROI) within the liver areas.

### Treatment trial

#### The sensitivity of CD90^+^ LCSCs, CD90^−^ Huh7 cells and Huh7 cells to magnetic hyperthermia

CD90^+^ LCSCs were cultured with DMEM/F12 containing hEGF, bEGF and B27 in low-attachment plates. To determine the sensitivities of CD90^+^ LCSCs, CD90^−^ Huh7 and Huh7 cells to hyperthermia, the viability and apoptosis of cells exposed to AMF for 10 min, 30 min and 1h were detected. These cells were incubated with TMs for 4h and then placed in an AMF to determine a defined temperature (44°C). Cells incubated with TMs not exposed to AMF were used as controls. After hyperthermia, cells were washed extensively to remove TMs and then replaced with fresh medium. The viability of CD90^+^ LCSCs, CD90^−^ Huh7 cells and Huh7 cells after 24 h was assessed by MTT assay. Apoptosis was determined by FACS Calibur flow cytometer using an Annexin V-FITC/PI apoptosis detection kit according to the manufacturer's instructions.

#### Targeted hyperthermia using CD90@TMs for CD90^+^ LCSCs

To compare the antitumor effect of CD90@TMs and TMs, the inhibition rate and apoptosis rate of CD90^+^ LCSCs was assessed by MTT and annexin V- FITC/PI affinity assays. Subsequently, CD90^+^ LCSCs were divided into seven groups: (1) a control group containing cells incubated with DMEM/F12, (2) an anti-CD90 MAb, (3) an anti-CD90 MAb, (4)TMs alone, unexposed to AMF, (5) CD90@TMs alone, unexposed to AMF, (6)TMs + AMF (7) CD90@TMs +AMF. All groups used 0.34 mg/mL Fe to reach a temperature of 44°C. Cells of group 3, 4, 5, 6 were incubated with TMs or CD90@TMs for 1h and hyperthermia groups were exposed to AMF for 1 h. Cell ultrastructure changes and the distribution of the Fe_3_O_4_ at the cellular level were observed by TEM. Firstly, the tumor cells were fixed in 2.5% glutaraldehyde and 1% osmium tetroxide in turn. After dehydration in graded ethanol solutions, the cells were embedded in SPI-Pon 812 (structure Probe, Inc; West Chester; USA). Ultrathin sections, stained negative by uranyl acetate and lead citrate were studied with TEM.

#### Effect of targeted hyperthermia on the characteristics of CD90^+^ LCSCs

There is no established method of determining the effects of hyperthermia on CSCs. Therefore, we investigated the characteristics of CSCs, including drug resistance, colony formation and invasion, as well as tumorigenic ability. CD90@TMs and TMs mediated hyperthermia were chosen to detect the effect of target hyperthermia and non-target hyperthermia to the characteristics of CSCs. Trypan blue staining was used as an index of cell activity prior to the experiment using the same number of cells as described previously. Tumor volume was calculated every day for 70 days with the following formula: tumor volume = long diameter × short diameter ^2^/2.

#### Effect of targeted hyperthermia on CD90^+^ LCSCs bearing mice

When the tumor volume reached about 600 mm^3^, CD90^+^ LCSCs-bearing mice were randomly divided into three groups: control group; TMs group (tumors injected with TMs and exposed to AMF); CD90@TMs group (tumors injected with CD90@TMs and exposed to AMF). Each group contained six mice. After 24 h, the hyperthermia groups were placed on an AMF (f = 200 kHz; I = 20A) for 60 min every other day. The maximal temperature of the rectal tissue did not exceed 40°C. Seven days later, all of the mice were euthanized, and then weighed and sectioned with HE staining. The cell apoptosis rate *in vivo* was determined by TUNEL assay according to manufacturer's instructions (Roche, Pleasanton, CA, USA). To assess the fraction of apoptotic cells, the count of TUNEL-positive cells was calculated from five sections. The expression rate of CD90 in the tumor was also detected by IHC staining. The inhibition rate of tumor volume was calculated as (1− volume of experimental group/volume of control groups) ×100%. The rate of inhibition of tumor mass was calculated as follows: (1-weight of experimental group/weight of control groups) ×100%. Proteins of different groups were extracted and quantified after treatment. Protein extracts (40 μg) were then separated by SDS-PAGE on 12% polyacrylamide gels and transferred to polyvinylidene fluoride membranes. Blots were blocked in 5% non-fat milk and incubated with anti-Bcl-2 (1:1000, Zhong Shan Golden Bridge Biotechnology, China), anti-Bax (1:1000, Zhong Shan Golden Bridge Biotechnology, China), and β-actin (1:10000, Sigma, USA) antibodies at 4°C overnight. Secondary antibody (1:10000, Thermo, USA) was incubated for 1 h and results visualized with SuperSignal^®^ West Pico Chemistry Luminescent Substrate (Thermo, USA). All the experiment repeated three times.

### Statistical analysis

Values represent means ± standard deviation (SD). The data were analyzed using the SPSS 16.0 software. A *p* value of < 0.05 was considered to indicate significance. A *p* value of >0.05 was considered to indicate no significance.

## Abbreviations

HCC: hepatocellular carcinoma
BCRP1: breast cancer resistance protein 1
MGMT: O (6)-methylguanine-DNA methyltransferase
MPS: mononuclear phagocytic system
CD90: cluster of differentiation 90
PEG_2000_-DSPE: 1,2-distearoyl-sn-glycero-3-phosphoethanolamine-N-[methoxy(polyethylene glycol)-2000]
TMs: thermosensitive magnetoliposomes
LCSCs: liver cancer stem cells
AMF: alternating magnetic field
MACS: magnetic-activated cell sorting
TEM: transmission electron microscope
FTIR: fourier translation infrared spectroscopy
Rh: Lissamine rhodamine B
DSC: differential scanning calorimeter
DMEM/F12: Dulbecco's Modified Eagle Media: Nutrient Mixture F-12
PI: propidium iodide
FITC: fluorescein isothiocyanate isomer I
MTT: 3-(45)-dimethylthiahiazo (-z-y1)-35-di- phenytetrazoliumromide
TUNEL: terminal deoxynucleotidyl transferase-mediated dUTP-biotin nick end labeling
HE: haematoxylin-eosin
FACS: flow activated cell sorting
NOD/SCID: nonobese diabetic/severe combined immunodeficien
IHC: immunohistochemical.
